# Pulmonary arterial sarcoma: A case report

**DOI:** 10.1097/MD.0000000000037194

**Published:** 2024-02-23

**Authors:** Yin Wang, Chunyan Rong, Jingwei Liu, Xuhan Liu, Weihua Zhang

**Affiliations:** a Department of Cardiology, First Hospital of Jilin University, Changchun, China.

**Keywords:** pulmonary artery sarcoma, pulmonary embolism, pulmonary endarterectomy, undifferentiated pleomorphic sarcoma

## Abstract

**Rationale:**

Pulmonary artery sarcoma (PAS) is a rare malignant tumor primarily originating from the pulmonary artery’s intima or subintima. Approximately one-third of cases are classified as undifferentiated type. Its clinical manifestations lack specificity, dyspnea is the main symptom but can also present with chest pain, cough, hemoptysis, and other discomforts, making it prone to misdiagnosis as pulmonary embolism (PE).

**Patient concerns:**

A 50-year-old woman was admitted to the hospital with “dyspnea for more than 3 months, aggravated for 2 days,” and computed tomography pulmonary angiography suggesting “bilateral multiple pulmonary embolisms.”

**Diagnoses:**

The patient was initially misdiagnosed as PE, and was later definitively diagnosed as undifferentiated pleomorphic sarcoma of the pulmonary artery by pathologic biopsy.

**Interventions and outcomes:**

The patient was initially treated with anticoagulant therapy, but her dyspnea was not relieved. After that, she underwent positron emission computed tomography (PET-CT) and other investigations, which suggested the possibility of PAS, and then she underwent pulmonary endarterectomy to remove the lesion, which relieved her symptoms and was advised to seek further medical attention from the Department of Oncology and Department of Radiotherapy.

**Lessons:**

PAS can be easily misdiagnosed as PE. If a diagnosis of PE is made, but anticoagulation or even thrombolytic therapy proves ineffective, and there is no presence of PE causative factors such as deep vein thrombosis in the lower extremities, or D-dimer levels are not high, one should be cautious and consider the possibility of PAS.

## 1. Introduction

Pulmonary artery sarcoma (PAS) is a sporadic malignant mesenchymal tumor originating from the pulmonary arteries, the etiology and pathogenesis are still unclear, with a reported incidence of 0.001% to 0.030%_._^[1,2]^ Mandelstamm first discovered and reported PAS by autopsy in 1923, and now there are less than 450 cases reported in the domestic and international literature.^[1,3–5]^ Clinical manifestations and imaging examinations of PAS lack specificity, often manifested as dyspnea, chest pain, cough, hemoptysis, syncope and other symptoms, and easily misdiagnosed as pulmonary embolism (PE) and pulmonary hypertension^[3,4]^ and pathological biopsy is the gold standard for the confirmation of the diagnosis of PAS.^[6,7]^ PAS is characterized by high metastasis, high mortality, and high misdiagnosis,^[2]^ and treatment can be divided into surgery, chemotherapy, radiotherapy, and immunotherapy, with surgery being the mainstay. It has been reported in the literature that the 1-year and 2-year survival rates after surgery are 22% and 7%, respectively.^[6,7]^ In this paper, we report a case of PAS misdiagnosed as PE admitted to the First Hospital of Jilin University and review the related literature.

## 2. Case report

**Figure 1.**
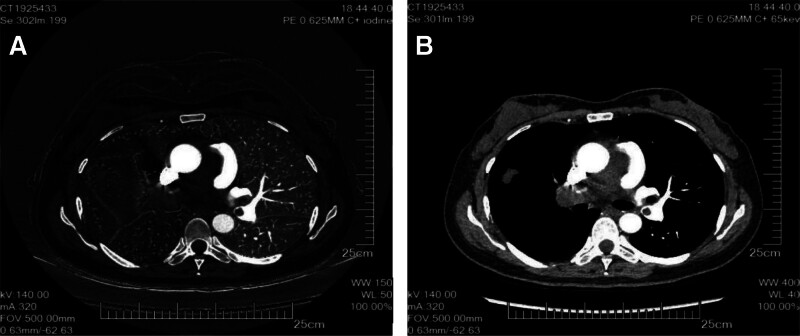
CTPA window and mediastinal window both show filling defects of the main pulmonary artery trunk and right and left pulmonary artery trunks A: pulmonary window; B: mediastinal window. CTPA = computed tomography pulmonary angiography.

**Figure 2.**
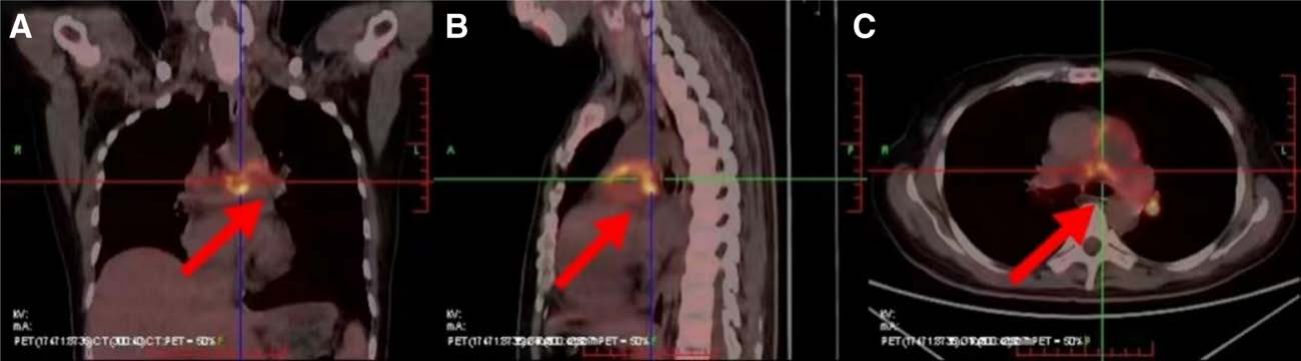
PET-CT whole-body imaging in coronal, sagittal, and cross-sectional views shows limited nodular metabolic increase from the main pulmonary artery trunk to the bifurcation of the right and left branches A: Coronal; B: Sagittal; C: Cross-sectional views. PET-CT = positron emission computed tomography.

The patient, a 50-year-old female, was admitted to the hospital’s emergency department with a suspected diagnosis of “PE.” She experienced dyspnea for more than 3 months, which had worsened over the past 2 days. The patient had initially developed dyspnea, low-grade fever, cough, and sputum after being infected with the novel coronavirus 3 months ago but had not received any treatment. Two days prior to admission, her symptoms worsened, and computed tomography pulmonary angiography (CTPA; Fig. 1) performed at the Second Hospital of Jilin University confirmed the presence of “bilateral multiple pulmonary artery embolism.” Subsequently, she was transferred to our hospital for further management. Physical examination: the body temperature was 36.4°C, the heart rate was 96 beats/min, blood pressure was 104/46 mm Hg, and breathing was 20 beats/min.

jugular venous distension. The heart border was slightly enlarged to the right, P2 > A2, grade 4/6 systolic wind-like murmur and right ventricular third heart sound could be heard in the tricuspid valve auscultation area, grade 4/6 systolic jet-like murmur was heard in the pulmonary valve area, and the hepatic jugular venous regurgitation sign was positive. Laboratory tests: B-type urinary natriuretic peptide: 1310.0 pg/mL, D-dimer: 1290 ng/mL; blood gas analysis: pH: 7.49, pCO2: 28 mm Hg, pO2: 67 mm Hg. There were no significant abnormalities in the thrombosis indexes of ultrasound of veins of both lower limbs, liver and kidney functions, tumor markers, ANA series, and anticardiolipin antibodies. Electrocardiogram (ECG) suggests ECG suggests: sI, qIII, tIII II, III, avF, T-wave flattening and inversion in leads V1 to V4. Cardiac ultrasound: 65% ejection Fraction, enlarged right atrium and right ventricle (right atrium 54 mm × 56 mm, right ventricle anterior-posterior diameter 35 mm, right ventricle transverse diameter 43 mm), estimated pulmonary artery systolic blood pressure 97 mm Hg, inferior vena cava collapsed > 50%, TAPSE = 18 mm (Table 1). Patient the heart rate was 96 beats/min, blood pressure was 104/46 mm Hg, SO2:95%, simplified Pulmonary Embolism Severity Index score 0, so the patient was diagnosed with acute PE (medium-low risk group), pulmonary heart disease, and cardiac function class III upon admission. Despite receiving symptomatic supportive treatments such as anti-infection (intravenous injection of ampicillin sodium 3 g every 12 hours), cardiac load reduction, diuresis (injection of recombinant human brain natriuretic peptide 0.5 mg), and anticoagulation (subcutaneous injection of enoxaparin sodium 4000 Axa IU every 12 hours), there was no improvement in the patient’s condition. Therefore, the patient was referred for a thoracic enhancement magnetic resonance examination, which revealed abnormal enhancement in the main trunk of the pulmonary artery and the right and left pulmonary artery trunks, suggestive of pulmonary artery sarcoma with surface embolism. Additionally, positron emission computed tomography (Fig. 2) showed localized nodular-like metabolic activity along the main trunk of the pulmonary artery to the bifurcation of the right and left branches, raising the possibility of malignancy (sarcoma?). Both findings indicate a high likelihood of malignant intravascular occupation in the pulmonary region. As a result, the patient was transferred to a higher-level hospital where they underwent partial resection of the pulmonary artery tumor and affected sections of the pulmonary artery. During the surgery, it was observed that the tumor had invaded the pulmonary valve, aortic valve, main trunk of the right and left pulmonary arteries, and some branches of the lobe-segmental level pulmonary arteries. The surgical procedure involved a complete resection of the tumor and reconstruction of the right and left main pulmonary arteries. Microscopic findings: the tumor cells are spindle shaped and there is a tumor in the wall of the pulmonary artery measuring 6.5 × 3 × 2.3 cm, of which the tumor size is 2.6 × 2.5 × 2.3 cm, the section is gray, grayish-yellow, solid and tough. Cellular anisotropy was evident and nuclear division was common. Immunohistochemical results: Ki-67 (index 80%), SMA (localized+), Desmin (-), MDM2 (+), BcL2 (-), CD31 (-), CD34 (-), MvoD1 (-), AE1/AE3 (-), ST AT6 (-), EMA (-), S-100 (-), Calretinin. Postoperative pathologic biopsy confirmed an undifferentiated pleomorphic sarcoma of the pulmonary artery.

**Table 1:** The significant laboratory test results at the first admission.

Inspection item	Values	Unit	Reference range
Temperature	36.4	°C	36.0–37.0
Blood pressure	104/46	mm Hg	90/60–140/90
Heart rate	96	times/min	60–100
Respiratory frequency	20	times/min	12–20
PH	7.49		7.35–7.45
PO2	67	mm Hg	83–108
PCO2	28	mm Hg	35–48
Blood oxygen saturation	95	%	93–98
BNP	1310.0	pg/mL	0–100
Troponin I	<0.05	ng/mL	0–0.05
WBC	12.7	10^9/^L	3.50–9.50
HB	98	g/L	115–150.
PLT	328	10^9/^L	125–350
CRP	68.91	mg/L	0–1.0
PTA	76	%	80–120
ALB	28.3	g/L	40.0–55.0
ATIII activity	55	%	80.0–130.0
Protein S activity	81.3	%	63.5–149.0
Protein C activity	54	%	70–140
FBG	5.59	g/L	1.8–4.0
D-dimer	1290	ng/mL	100–600
EF	65	%	50–70
Right atrium	54 × 56	mm	34–49 × 29–45
Right ventricular anteroposterior diameter	35	mm	25–40
Right ventricular transverse diameter	43	mm	25–40
Ultrasound of lower extremity veins	No thrombosis		

Rows 23-26 in Table 1 represent cardiac ultrasound.

ALB = Albumin, ATIII = activity Antithrombin III, BNP = Brain Natriuretic Peptide, CRP = C-reactive protein, EF = ejection Fraction, FBG = Fibrinogen, HB = Hemoglobin, PCO2 = Partial Pressure of Carbon Dioxide, PH = the potential of hydrogen, PLT = Platelet, PO2 = Partial Pressure of Oxygen, PTA = Prothrombin Time Activity, WBC = White Blood Cell.

## 3. Discussion

PAS is a rare type of malignant tumor that develops in the pulmonary artery or pulmonary valve. The World Health Organization classifies PAS into 2 types: wall sarcoma, which is mainly smooth muscle sarcoma, and intimal sarcoma.^[8]^ PAS usually presents with subtle symptoms, making it difficult to diagnose. It has a poor prognosis, with a median survival period of 17 months, PAS commonly affects individuals between the ages of 40 and 60, with a male-to-female ratio of 1:2.^[3,8,9]^ The exact causes and underlying mechanisms of PAS are still unknown. Hagwat et al^[1,10,11]^ have suggested that PAS may originate from multipotent mesenchymal cells with the ability to differentiate in multiple directions, potentially derived from the embryonic cardiac balloon myogenic material. The most common pathological type of PAS is undifferentiated sarcoma (34%), followed by fibrosarcoma (21%), smooth muscle sarcoma (20%), rhabdomyosarcoma (6%), mesenchymal histiocytoma (6%), intrachondral sarcoma (4%), angiosarcoma (4%), osteosarcoma (3%), and malignant fibrous histiocytoma (2%).^[11,12]^ Among these, smooth muscle sarcoma generally has a better prognosis, while rhabdomyosarcoma has the poorest prognosis, typically resulting in death within 3 months after surgery. Undifferentiated tumors fall in between in terms of malignancy.^[11,12]^ In a specific case, the pathological diagnosis was undifferentiated pleomorphic sarcoma of the pulmonary artery, accounting for approximately one-third of all PAS, this type of sarcoma is characterized by a large mass originating from the main pulmonary artery outside the lungs, often growing into the blood vessel lumen as a polypoid mass.^[11,12]^ Dewaele et al^[6,13]^ suggests that the development of PAS may be associated with the presence of endosomal sarcomas and the overexpression of certain genes, such as platelet-derived growth factor receptor, MDM2, CKD4, SAS, and sustained activation of the epidermal growth factor receptor.^[13,14]^ Inhibition of MDM2 has been proposed as a potential therapeutic target for patients with MDM2(+).^[13,15]^ It is important to note that these findings are based on limited research, and further exploration is needed to fully understand the specific pathogenesis of PAS.

The clinical manifestations of PAS are often atypical, with dyspnea being the most common symptom.^[1,2]^ In this particular case, the patient presented with dyspnea. Upon examination, her vital signs were stable, and a cardiac murmur was audible. The patient was also found to have tricuspid valve insufficiency and pulmonary valve stenosis, which were attributed to the enlargement of the right atrium and right ventricle.^[16,17]^ Laboratory tests revealed elevated levels of brain natriuretic peptide, while troponin levels were normal. The ECG displayed specific abnormalities such as sI, qIII, tIII, II, III, and avF, as well as T-wave hypoplasia and inversion in leads V1 to V4. These findings suggested insufficient myocardial blood supply, myocardial injury, and cardiac enlargement.^[2,18].^

The cardiac ultrasound revealed an enlarged right heart and pulmonary hypertension. Additionally, CTPA suggested the possibility of PE. The patient’s pulse rate was 96 beats per minute, blood pressure was 104/46 mm Hg, and SO_2_ was 95%. According to the simplified Pulmonary Embolism Severity Index score, the patient was categorized as 0, and the preliminary diagnosis was PE (medium-low risk group).^[16]^ Initial treatment included anticoagulation therapy and other symptomatic treatments. However, the patient’s condition did not improve. It is commonly observed that patients with PE often have concurrent lower extremity venous thrombosis, and anticoagulation therapy is effective in low and intermediate-risk patients.^[16]^ However, in this particular case, the patient had normal findings on lower extremity venous ultrasound and no other high-risk factors for the development of PE. Despite receiving standardized anticoagulation therapy, the patient’s dyspnea did not significantly improve. We carefully reviewed CTPA findings and observed that the occupying space predominantly affected the main pulmonary artery trunk. The enhancement pattern was heterogeneous, rather than a uniform density filling defect, and there were notch-erosion-like changes in the pulmonary artery wall. These findings raised strong suspicion for an aggressive malignant tumor, rather than PE. To further investigate, we conducted an enhanced magnetic resonance of the chest and PET/CT scans. These tests revealed limited nodular areas with increased metabolic activity from the main trunk of the pulmonary artery to the bifurcation of the right and left branches. Malignant possibilities, such as sarcoma, could not be ruled out based on the imaging results. Considering these findings, the patient was initially diagnosed with PAS. Pathological biopsy is considered the gold standard for diagnosing this disease, and the patient underwent this procedure.^[10–12]^ Subsequently, the patient was referred to a higher-level hospital for pulmonary endarterectomy. The postoperative pathology confirmed the presence of undifferentiated pleomorphic sarcoma of the pulmonary artery, providing a definitive diagnosis of pulmonary artery sarcoma.

Both PE and PAS can lead to obstruction of blood flow in the pulmonary artery, which causes pulmonary hypertension, increases the load on the right ventricle, and finally enlarges the right atrium ventricle, resulting in abnormalities of the patient’s electrocardiogram, cardiac ultrasound, etc.,^[8,19]^ therefore it is very difficult to differentiate PE from PAS by conventional auxiliary examinations.^[6,8,11]^ CTPA is a commonly used adjunctive test for the diagnosis of PAS,^[8–10]^ and in patients with PAS, the main manifestations of CTPA are dilatation of the pulmonary arteries, filling defects in the lumen, narrowing of the lumen, and occupancy of the pulmonary arteries, which may be manifested as an “eclipsed wall sign,” but is atypical of early PAS.^[8–10]^ computed tomography (CT) and magnetic resonance imaging are probably the most useful tests for differentiating between tumor and thrombosis.^[8,19]^ Magnetic resonance imaging shows moderate to low signal on T1W and high signal on T2W and exhibits heterogeneous enhancement, whereas PE shows high signal on T1W and T2W and enhancement scans do not intensify, but the specificity of this test is low.^[19]^ Increased uptake of 18F-FDG on PET/CT can be helpful in the diagnosis of PAS.^[8,19,20]^ Studies have reported that PET/CT can differentiate between PAS and PE based on the maximum standardized uptake value (SUV max) (7.63 ± 2.21 for PAS and 2.31 ± 0.41 for PTE).^[8,19,20]^ It has also been suggested that an SUV max threshold of 3.5 provides 100% sensitivity, specificity, and accuracy in distinguishing PAS and PE)^[20]^ (Table 2). In our patient, the SUV max value of 6.6 further supports the diagnosis of PAS.

**Table 2:** Differentiation of PAS from PE.

	PAS	PE
Lesion site	It occurs most often in the main pulmonary trunk and bilateral pulmonary trunks, more often on the right than on the left, and can extend to the pulmonary valve and right ventricular outflow tract, main pulmonary artery	Most common in the right lung, both lower lungs and peripheral pulmonary arteries
Morphology of lesions	Complete shape, swollen growth, proximal projection or lobule, distal expansion or grapelike shape and enhanced heterogeneity	The lesion is proximally flattened or cupped
Presence of risk factors for thrombosis	None	Mostly
D-dimer	Mostly normal	Mostly elevated
Anticoagulation or thrombolysis	Null	Validity
Ultrasound of both lower extremity veins	Mostly normal	May have deep vein thrombosis
Echocardiography	a. The surface of the tumor is irregular, mostly heterogeneous with strong echoes and peripheral echoes, which are paged or polypoid; b. The tumor has a certain degree of mobility in the early stage when it has not yet filled the vascular lumen; c. It can find blood flow signals in the tumor.	a. Homogeneous echogenic changes, no envelope, more fixed morphology b. Thrombus without blood flow signal
CTPA	Usually involves the main pulmonary trunks and pulmonary valves, with expansive growths and proximal “eclipsed wall sign”	It often involves the right and left pulmonary arteries, both lower lungs and the peripheral pulmonary arteries, with a proximal saddle-shaped flat or cup-shaped thrombus, and no “eclipsed wall sign”
18F-FDG PET/CT SUVmax 3.5	More than 3.5	Less than 3.5
Combined lung or mediastinal lesions	Mostly	None
Pathological biopsy	Can detect tumor cells	Tumor cells cannot be detected
Thrombolytic effects of anticoagulants	Null	Validity

PAS = pulmonary artery sarcoma, PE = pulmonary embolism.

PAS is associated with low specificity, a high rate of metastasis, misdiagnosis, and mortality.^[14,15]^ At the time of diagnosis, approximately 50% of patients already have lung metastases, and 16% have distant metastases.^[3,11,12]^ In this case, the patient had scattered small nodules in the lungs with a partial increase in metabolic activity on positron emission computed tomography, suggesting pulmonary metastases. Treatment options for PAS include surgery, chemotherapy, radiotherapy, and immunotherapy.^[3–6]^ Surgical resection of the lesion is the preferred treatment, which may involve lung or lobectomy, pulmonary endarterectomy, or ultrasound bronchoscopy-guided transbronchial needle aspiration biopsy.^[3–6]^ However, postoperative complications such as hemorrhage, infection, respiratory embolism, and recurrence are common in adult patients.^[3–5]^In this case, the patient underwent partial resection of the pulmonary artery tumor and pulmonary artery. Postoperatively, the patient was advised to seek further consultation in oncology and thoracic surgery and to undergo regular follow-up at 1 month, 3 months, 6 months, 1 year, 2 years, and 5 years to monitor their condition.

Pulmonary endarterectomy is associated with a higher survival rate compared to isolated tumor resection.^[3–6]^ The prognosis for patients with pulmonary artery sarcoma is influenced by factors such as complete tumor resection, the type of pathology, the patient’s age, and the presence of metastases.^[3,4,9]^ Postoperative radiotherapy and chemotherapy can have a positive effect on the treatment of pulmonary artery sarcomas^,[6,13]^ but there is no standard treatment protocol. Some studies suggest that chemotherapeutic agents like isocyclophosphamide, epirubicin, and adriamycin may be effective.^[7,11–14]^ Furthermore, combining adriamycin and isocyclophosphoric acid amine in chemotherapeutic regimens has shown efficacy regardless of the histological subtype of these tumors.^[7]^ In conclusion, for patients with PAS who are not suitable for surgical resection or have a high risk of postoperative recurrence, a combination of multiple therapies should be considered whenever possible.^[5–7,11]^

In summary, PAS is clinically rare and easily confused with PE. Through the demonstration of the clinical diagnosis and treatment process in this case, we need to be highly vigilant for PAS in patients diagnosed with PE if thrombolytic or anticoagulant therapy is ineffective, there is a lack of causative factors for PE such as deep vein thrombosis of the lower extremities, or D-dimer is not high.

## Author contributions

**Data curation:** Yin Wang.

**Formal analysis:** Chunyan Rong.

**Funding acquisition:** Weihua Zhang.

**Methodology:** Xuhan Liu.

**Project administration:** Jingwei Liu.

**Resources:** Chunyan Rong, Weihua Zhang.

**Software:** Jingwei Liu.

**Supervision:** Chunyan Rong.

**Validation:** Xuhan Liu.

**Writing – original draft:** Yin Wang.

**Writing – review & editing:** Xuhan Liu, Weihua Zhang.
